# Supplementary material for: Real-time interleaved spectroscopic photoacoustic and ultrasound (PAUS) scanning with simultaneous fluence compensation and motion correction
Source: Nat Commun. 2021 Jan 29;12:716. doi: 10.1038/s41467-021-20947-5 (PMC7846772; doi:10.1038/s41467-021-20947-5)
Supplement: Supplementary file 1 — Supplementary Information [file 41467_2021_20947_MOESM1_ESM.pdf]

Supplementary information

to the paper

Real-time spectroscopic photoacoustic ultrasound (PAUS) scanning with simultaneous fluence compensation and motion correction

Geng-Shi Jeng[1,2], Meng-Lin Li[3,4], MinWoo Kim[1], Soon Joon Yoon[1], John J. Pitre Jr.[1], David S. Li[4], *Ivan Pelivanov[1] and Matthew O'Donnell[1]

[1]Department of Bioengineering, University of Washington, Seattle, WA 98195, USA

[2] Institute of Electronics, National Chiao Tung University, Hsinchu, Taiwan

[3]Department of Electrical Engineering, Institute of Photonics Technologies, National Tsing Hua University, Hsinchu, Taiwan

[4]Institute of Photonics Technologies, National Tsing Hua University, Hsinchu, Taiwan

[5]Department of Chemical Engineering, University of Washington, Seattle, WA 98195, USA

<u>Supplementary Note 1</u>. Diode-pumped, wavelength-tunable laser

A unique (see Specification sheet below), high pulse repetition rate (from single shot to 1000 Hz), wavelength tunable (700-900 nm) laser (Laser-Export, Russia) was designed and built especially for the fast-sweep PAUS system. Its principle is based on pumping the Ti:Sapphire laser head with the second harmonic of the Nd:YLF diode-pumped laser and using an acousto-optic filter for fast selection of wavelengths. All laser components were assembled within a compact block.

The laser can emit pulses of about 1 mJ energy at a 1000 Hz repetition rate, with wavelength switching times less than 1 ms for any arbitrary order of wavelengths (i.e., wavelength does not need to be sequentially stepped between bounds). Every laser pulse in a sequence can be at a different wavelength without sacrificing the kHz repetition rate. The number of pulses per wavelength in a sequence and energy of every pulse in percent from the maximum can be pre-set. The pulse repetition rate can be set in the range 1 – 1000 Hz for both single- and multi-wavelength operation. Other laser parameters are listed in the specification sheet below.

LASER SPECIFICATION

Wavelength tuning range .... 700 - 900 nm
Mode of Operation .... Q-switched
Average Pulse Energy at 1 kHz at 700 nm .... > 500 μJ
Average Pulse Energy at 1 kHz at 900 nm .... > 600 μJ
Max. Average Pulse Energy at 1 kHz in the range of 780 - 820 nm .... 1000 μJ
Beam Profile .... TEM_{00}
Beam Diameter ($1/e^2$, at output aperture) .... 0.9 mm
Pulse duration at the wavelength of maximum energy .... 8-13 ns
Pulse-to-Pulse Stability (StdDev/Mean, 1 kHz, at nominal pulse energy)
    at set wavelength regime .... <8 %
    at varying wavelength regime (for pulses of the same wavelength) .... <10 %
Polarization Linearity .... > 100:1
Range of Pulse Repetition Rate:
    Ext. Triggering .... 0 - 1 kHz
    Int. Triggering: through RS-232 .... 0 - 1 kHz
    without PC .... 1 ± 0.01 kHz
Trigger Pulse Fall Edge (or Q-switcher trigger pulse) to Laser Pulse
    Delay (the value within the range) .... 300 ± 250 ns
    Jitter .... < 8 ns
Dimensions (maximum linear size):
    Laser Head .... 250 mm
    Power Supply .... 300 mm
Weight:
    Laser Head .... <6 kg*
    Power Supply .... < 7.5 kg*
Wavelength Switching Rate per Step .... <1 ms
Wavelength Tuning Control .... preset mode, PC mode
Remote Control of Laser Parameters:
Pumping ON/OFF, Ext./Int. Triggering, pulse repetition rate, pulse energy .... via RS-232
DTR via RS-232 .... 4800 bit/s
Operating Temp/Humidity Range .... +15 to + 35 ºC /up to 80% non-condensing
Operating Voltage .... 24 ± 10% VDC
Laser Class IV
Compliance .... CE, RoHS

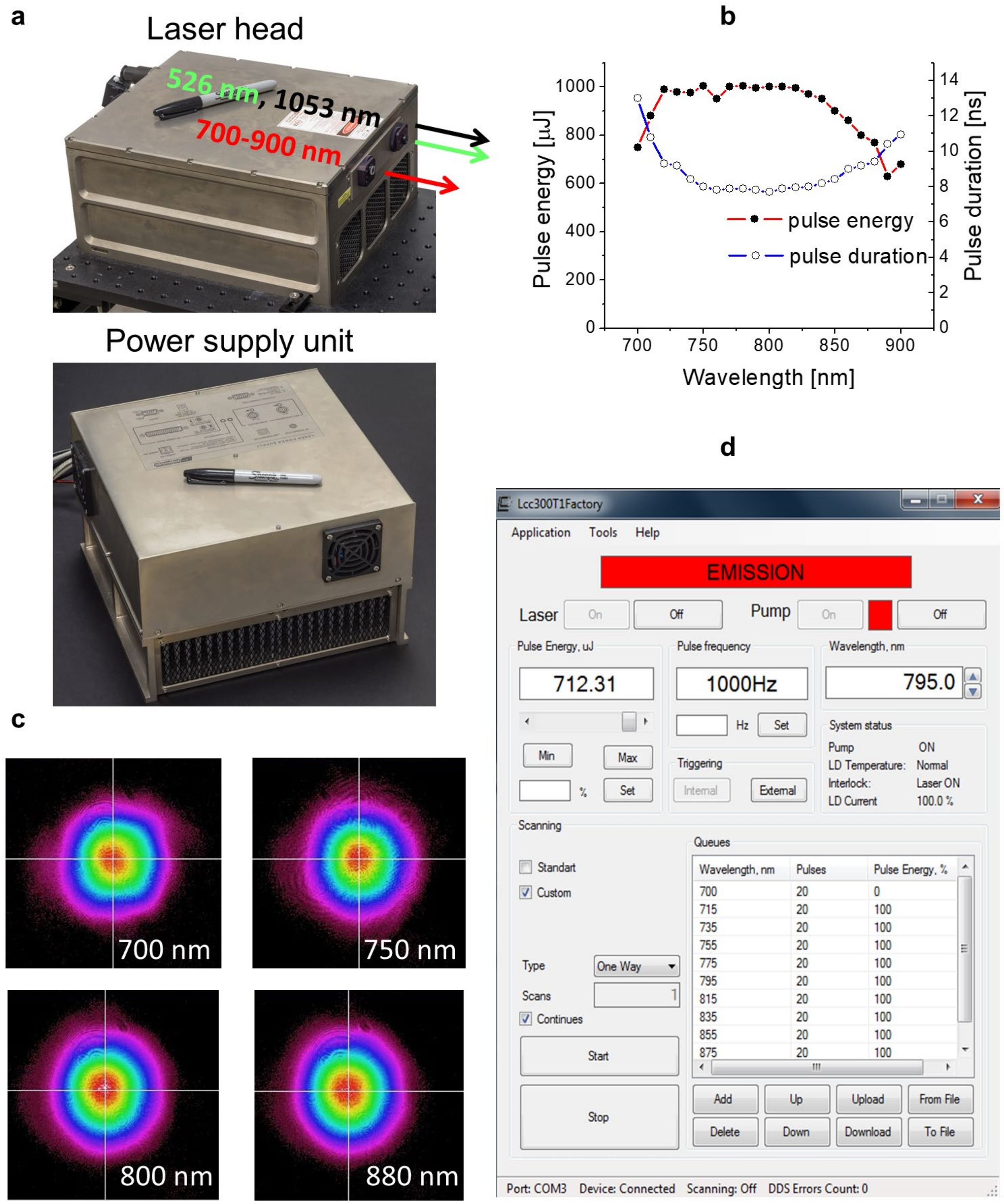

Supplementary Fig. 1: Diode-pumped, kHz rate, wavelength-tunable Ti:Sa laser. a, photograph of the laser; **b**, wavelength dependence of laser emission energy and pulse duration; **c**, beam profiles; **d**, example of a pulse sequence (implemented continuously with no delays for wavelength or regime switching).

Supplementary Note 2. PAUS transmission/detection head

The PA probe head combines a commercial 15-MHz, 128-element US linear-array probe with a pitch of 0.1 mm (Vermon, France) with 20 fibers spanning the elevational extend of the array as two fiber arrays (Supplementary Fig. 2a). Each fiber array (i.e., 10 fibers) has an interval of 1.5 mm, spanning 13.5 mm over the whole US lateral image range (i.e., 12.8 mm). Two fiber arrays are separated by a distance of 12 mm, and tilted symmetrically relative to the US image plane with an angle of 35°. Consequently, beams generated from each fiber array intersect on the same image plane at a depth of 8 mm around the transducer's elevational focus (Fig. 2b). The impulse and frequency responses of the whole system, including the probe to PA acquisition, were measured by receiving the PA signal at a single channel from a point-like absorber (i.e., human hair), as shown in Fig. 2d.

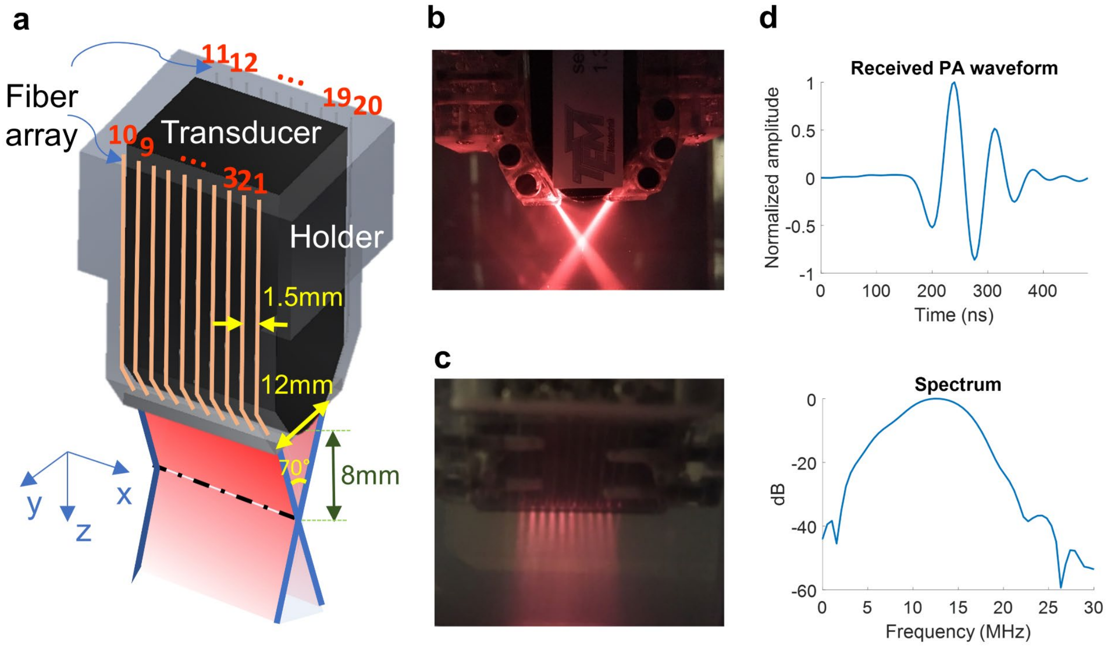

Supplementary Fig. 2: Integrated PA probe with 20 fibers. **a,** 3-D schematic view. **b,** photograph of the integrated probe viewed in the y-z plane. **c,** photograph viewed in the x-z plane. **d,** Measured PA waveform and frequency response from a point-like absorber (i.e., human hair).

Supplementary Note 3. **Schematic diagram for real-time fast-sweep spectroscopic PAUS imaging**

Ten different laser wavelengths form a spectroscopic scan cycle (see Supplementary Fig. 3a). Motion among all 10 B-modes is estimated. The estimated displacement is then applied to all PA sub-images. By identifying strong absorbers in a PA image, wavelength-dependent optical fluence is estimated using the diffusion approximation. The estimated fluence is then compensated for all PA sub-images over the 10 different wavelengths. A wavelength-compounded image (called Σλ-PA) is then produced by coherent summation over all 10 wavelengths. Quantitative PA spectroscopy is performed pixel-by-pixel. To accurately estimate a component spectrum, the noise level is estimated by turning off the laser at 700 nm and then subtracted from the measured spectrum. The compensated, noise-subtracted spectrum is correlated with the reference spectrum (e.g., the absorption spectrum measured from a UV-VIS spectrophotometer). Finally, multiplying Σλ-PA by the correlated result produces component-weighted images. By shifting one-wavelength frame, another new spectroscopic image can be obtained simply using the latest 10 wavelengths at a 50 Hz frame rate, after appropriate motion and fluence compensation over this set of measurements. Note that a persistence filter can be applied to produce smooth spectroscopic results at a reduced rate but the spectroscopic frame is updated at 50 Hz.

Radio-frequency PAUS channel data for the Nth wavelength are acquired and transferred to the PC via PCI Express 2x16 lanes (data transfer rate > 5.6 GBs^{-1}) while processing channel data for the $(N-1)^{th}$ wavelength (see Supplementary Fig. 3b). Given 1 US B-Scan (64 scan lines for dual beam) and 20 PA sub-frames sampled by 2048 points and represented by 2 bytes, the total PAUS 128-channel data transfer time is 7.9 ms. In parallel, beamforming for the "last" PAUS frame is conducted using a graphics processing unit (GPU). The processing time to produce one B-Scan and 20 subimages is around 16.6 ms. This includes the time for transferring channel data from the CPU to GPU memory (around 4 ms). Motion estimation between two successive B-modes can be performed using pipeline processing after forming the B-mode, followed by motion compensation applied to 20 PA subimages. The processing time for both motion estimation and compensation is around 11 ms (see Supplementary Note 9). Fluence estimation is performed every 200 ms (i.e., 10 wavelengths) after applying motion compensation on the tenth wavelength PAUS image (i.e., 875 nm). It takes 23 ms to complete fluence estimation (Supplementary Note 6). However, because we intentionally disable the laser at the first wavelength (700 nm) for noise estimation, fluence compensation starts from the second wavelength (715 nm) before obtaining 9 wavelength fluence estimates. Overall, with parallel and pipeline processing, the processing time for the major algorithms mentioned above is less than 17 ms. See Supplementary Data and Software Library for the details of real-time implementation.

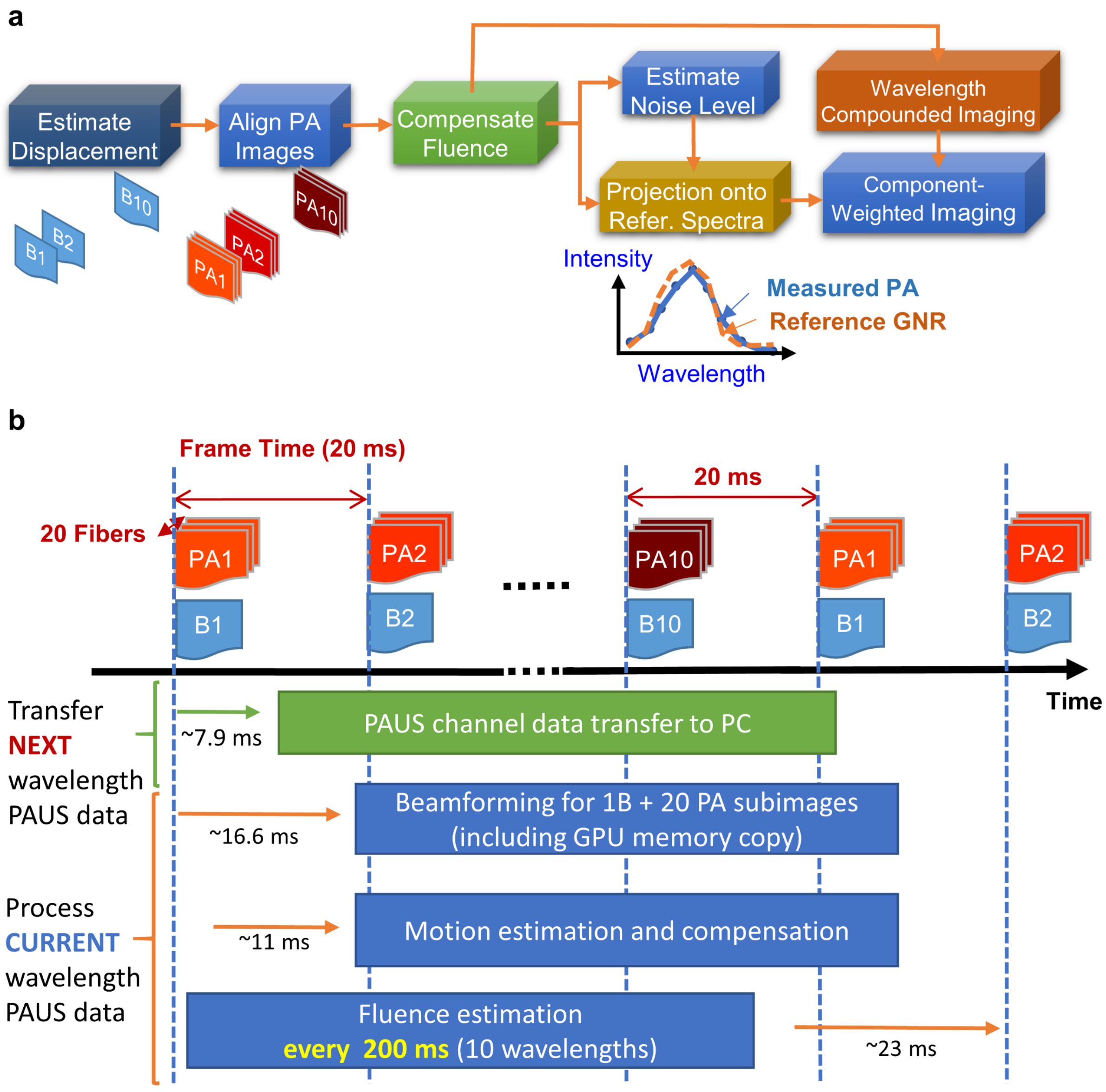

Supplementary Fig. 3: a, Flow chart for real-time fast-sweep spectroscopic PAUS imaging. **b,** Timing diagram for real-time fast-sweep spectroscopic PAUS imaging.

Supplementary Note 4. **Σλ-PA and component-weighted imaging**

Both wavelength-compounded PA (Σλ-PA) and component-weighted PA images are processed after performing motion correction and fluence compensation. As shown in Supplementary Fig. 4, Σλ-PA images are the coherent sum over all wavelength PA images (i.e., 9 wavelengths ranging from 715 to 875 nm used in this study). Component-weighted PA images are produced by correlating specific molecular constituent spectra with measured PA signal spectra. This is obtained by computing the correlation between the measured PA spectrum and the known spectrum of a specific molecular absorber. First, all 9 wavelength PA images are subtracted pixel-wise from noise estimates obtained by turning off the 700 nm laser emission. Next, for the i-th pixel, the normalized cross correlation (NCC) between the spectral estimate $x_i(\lambda)$ from N_λ wavelengths and the reference spectrum $y(\lambda)$ (i.e., UV-VIS measurements) is computed:

$$C_{x_i,y} = \frac{\sum_{k=1}^{N_\lambda}[x_i(\lambda_k) - \bar{x_i}][y(\lambda_k) - \bar{y}]}{\sqrt{\sum_{k=1}^{N_\lambda}[x_i(\lambda_k) - \bar{x_i}]^2}\sqrt{\sum_{k=1}^{N_\lambda}[y(\lambda_k) - \bar{y}]^2}}, \qquad \text{(S1)}$$

where $C_{x_i,y}$ is the NCC value for the i-th pixel. $\bar{x_i}$ and $\bar{y}$ represent the mean value of the estimated and reference spectrum over all N_λ wavelengths, respectively.

Using the NCC for a particular absorber, a weight is obtained at each pixel in the image for that absorber from a scaling function. In this study, a sigmoid was used to produce the weighting map for each absorber to minimize noise for low NCC and highlight regions of high NCC. For each NCC value *X*, the weighting result *Y* is given by

$$Y = \frac{1}{1 + e^{-a(X-b)}}$$
$$0 \leq X, Y \leq 1; \;\; 0 \leq a \leq \infty; \;\; 0 \leq b \leq 1 \;, \qquad \text{(S2)}$$

where *b* represents the NCC value corresponding to *Y* = 0.5 and *a* determines the slope of the function (Supplementary Fig. 4). This non-linear function can slightly distort quantitative information in pixels where multiple absorbers contribute to the signal, but it maintains quantitative accuracy in pixels dominated by a single absorber while suppressing noise in regions of low PA signal. In this study, *a* and *b* for GNR-weighted PA images are 50 and 0.82, respectively. a and b for needle-weighted PA images are 60 and 0.9, respectively. Finally, a component weighted PA image is produced for each molecular absorber by multiplying the appropriate weighting map with the Σλ-PA image on a pixel-by-pixel basis.

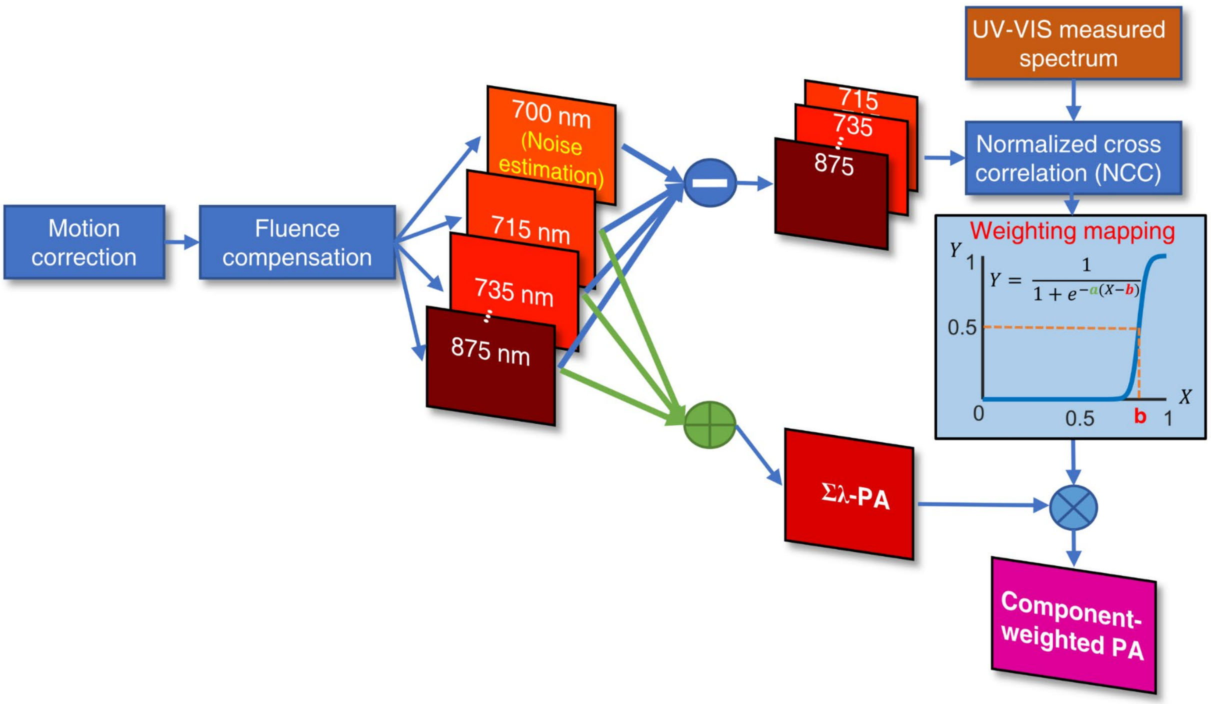

Supplementary Fig. 4: Signal processing flow chart for wavelength-compounded (Σλ-PA) and component-weighted PA imaging.

Supplementary Note 5. **Agent properties**

Gold nanorods solution. PEG-coated, 45 nm long, gold nanorods (GNRs) manufactured by NanoHybrids (Austin, TX, USA) were used. The properties are summarized in Supplementary Table 1. The measured UV-VIS absorption coefficient of the GNR solution is shown in Supplementary Fig. 5a. Given the size and aspect ratio of the GNR, the solution optical density is dominated by optical absorption.

Supplementary Table 1. Properties of gold nanorods used in this study

**Longitudinal Peak**	**776 nm**
**Longitudinal Optical Density**	51.0
**Peak Width at 80%**	68 nm
**Transverse Optical Density**	12.4 nm
**Longitudinal/Transverse**	4.1
**Particle Concentration**	3.9×10^{13} per ml
**Mass Concentration (Au)**	2.2 mg/ml
**Width**	11.4 ± 1.1 nm
**Length**	44.8 ± 3.4 nm
**Aspect ratio**	3.9
**Solvent**	De-ionized and ultrafiltrated water
**Particle Surface**	mPEG, 5kDa
**Zeta Potential**	-3.1 mV
**pH**	7.1

Black ink solution. Black ink solution (#44011, Higgins, MA, USA) was used as a strong absorber injected into the tube without dilution. The measured extinction spectrum is shown in Supplementary Fig. 5b where 30 µl black ink was diluted by 1 ml de-ionized water.

Prussian blue nanoparticles. Prussian blue nanoparticles were synthesized using methods adapted from Zhu *et al.* [1] and Ren *et al.* [2] To prepare the Prussian blue nanoparticles, stock solutions of 250 ml of 1 mM iron (III) chloride (Sigma-Aldrich, product number: 157740, CAS: 7705-08-0), 250 ml of 1 mM potassium ferrocynide (Sigma-Aldrich, product number: P3289, CAS: 14459-95-1), and 500 ml 0.5 mM sodium citrate solution (Sigma-Aldrich, product number: W302600, CAS: 6132-04-3) were prepared in pure 0.2 µm filtered de-ionized water. All stock solutions were brought to 60°C while stirred using a magnetic bar while on a hotplate. The sodium citrate solution was divided equally between the iron (III) chloride solution and the potassium ferrocynide solution and stirred until fully mixed. Finally, the potassium ferrocynide with sodium citrate solution was added to the iron chloride with sodium citrate solution, gradually forming a slightly green tinted blue solution. The final solution was stirred at 60°C for 1 minute before allowing the solution to cool to room temperature while continuously stirring. The particles were purified and concentrated by washing them four times with pure deionized water through a 30 kDa pressure-driven ultrafiltration system. The particles were concentrated to a peak optical extinction value of 56 cm^{-1} at 720 nm (Supplementary Fig. 5c).

Solution of Prussian blue mixed with 1% Intralipid and de-ionized water used in Fig. 3. We used 0.47 ml Prussian blue diluted by 380 ml de-ionized water prior to adding 1% Intralipid. The measured absorption coefficient of the mixed Prussian blue and water is shown in Supplementary

Fig. 5d. This spectrum is used to predict μ_{eff} of the final solution by incorporating the measured μ_s' of pure Intralipid in Ref. [3] (See Eqs. (12) and (13) in Ref. [3]). Accordingly, using least-squares fitting to Mie theory, the measured scattering coefficient (denoted as μ_s) and the anisotropy coefficient (denoted as g) of 10% Intralipid are [3]

$$\mu_s(\lambda) = 0.016\lambda^{-2.4} \quad (\pm 6\%)$$
$$g(\lambda) = 1.1 - 0.58\lambda \quad (\pm 5\%) \tag{S3}$$

respectively, where λ is the wavelength between 0.4 μm and 1.1 μm and μ_s is in $mL^{-1}Lmm^{-1}$. The uncertainties of both values are also indicated [3]. Using the relations $\mu_s' \triangleq \mu_s(1-g)$ and $\mu_{eff} \triangleq \sqrt{3\mu_a\mu_s'}$ (where μ_a denotes the absorption coefficient), the predicted μ_{eff} is shown in Fig. 3c.

Needle absorption spectrum. Needle-weighted PA imaging requires the reference needle absorption spectrum. For this purpose, it was estimated in de-ionized water by sweeping the wavelength from 715 to 875 nm (Supplementary Fig. 5e). The needle was 21 gauge with 0.82-mm outer diameter (21G1, BD, New Jersey, NJ, USA). The estimated spectrum shown in Supplementary Fig. 5e was used as the reference spectrum in Fig. 4.

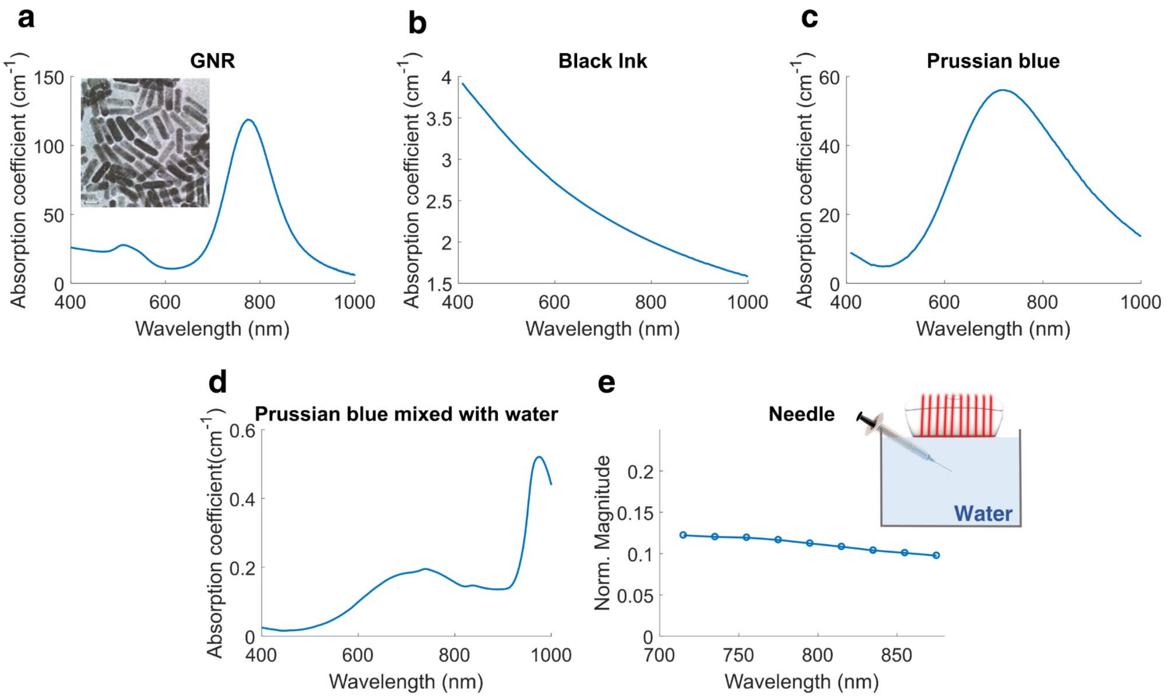

Supplementary Fig. 5: UV-VIS and PA results for all agents used in this study. **a,** GNR. **b,** Black ink. **c,** Custom-made Prussian blue nanoparticles. **d,** Solution used to study fluence compensation in Fig. 3 without adding Intralipid® (i.e., Prussian blue mixed with de-ionized water). **e,** Setup to measure the needle absorption spectrum in de-ionized water. The measured needle spectrum (normalized to the area under the spectrum) over the wavelength range of interest in this study.

Supplementary Note 6. Light-diffusion based laser fluence estimation in the fast-sweep geometry

PA Signal: For the i-th fiber, the reconstructed PA sub-image $S_i(\vec{r},\lambda)$ at position $\vec{r}$ and wavelength λ resulting from illumination of a short laser pulse onto an absorber inside tissue can be represented as

$$S_i(\vec{r},\lambda) = \Gamma\mu_a(\lambda)\Phi_i(\vec{r},\lambda)\ , \tag{S4}$$

where Γ is the Grüneisen coefficient, $\mu_a(\lambda)$ is the absorber's wavelength dependent optical absorption coefficient, and $\Phi_i(\vec{r},\lambda)$ denotes the optical fluence generated by the i-th fiber at this position and wavelength. By coherent summation over all PA sub-images for all fibers N_f, the resulting PA image $P(\vec{r},\lambda)$ is expressed as

$$P(\vec{r},\lambda) = \sum_{i=1}^{N_f} S_i(\vec{r},\lambda) = \Gamma\mu_a(\lambda)\sum_{i=1}^{N_f} \Phi_i(\vec{r},\lambda), \tag{S5}$$

where $P(\vec{r},\lambda)$ is simply proportional to the product of the local light absorption coefficient with the sum of laser fluences associated with integrated illumination from all 20 fibers. Hence, to accurately estimate $\mu_a(\lambda)$ from PA measurements, the fluence estimate $\Phi_i(\vec{r},\lambda)$ for every fiber and wavelength must be obtained.

Fluence model: Biological tissues are multi-component turbid media consisting of collagen fibers, connective tissue, fat and blood at different levels of oxygenation. Optical properties of each of these components are different [4, 5] and, thus, most biological tissues are optically micro-heterogeneous. However, if optical heterogeneities are distributed and their size is smaller than the photon transport mean free path $l_{tr} = 1/\mu_s'$ (typically ranging from 1 mm to 1 cm), light transport to depths exceeding a few l_{tr} can be characterized by global (or effective) optical parameters: light absorption μ_a and reduced scattering μ_s', i.e. a model of optically macro-homogeneous medium is considered. A recent summary of tissue optical properties can be found in [4].

The fluence $\Phi_i(\vec{r},\lambda)$ can be represented using the diffusion approximation (see below) to the light transport equation [6-9]. This approximation accurately models the energy distribution in a turbid medium at depths $\gtrsim$ the photon transport mean free path [10]. The solutions for plane, cylindrical and point sources have been presented in the literature [9-12]. Note that in our geometry, light sources are located at a 6 mm distance from the imaging plane. Thus, the diffusion approximation should be accurate for all points of the imaging plane when $\mu_s' \gtrsim 2\ \text{cm}^{-1}$.

Under the approximation of an optically macro-homogeneous medium, the steady-state diffusion equation for an optical source $I(\vec{r},\lambda)$ describing the optical fluence distribution in biological tissue at depths $\gtrsim l_{tr} = 3D = 1/\mu_s'$ reduces to [6-9]

$$\mu_a(\lambda)\Phi(\vec{r},\lambda) - D(\lambda)\,\nabla^2\Phi(\vec{r},\lambda) = I(\vec{r},\lambda)\ , \tag{S6}$$

where $D(\lambda)$ is the diffusion coefficient, assumed to be spatially invariant. The exact expression of D and its dependence on light absorption, scattering and anisotropy factor is still an open issue in the literature [9, 10, 13-15]. The most general expression can be found in Ref. [14]. However, in our case of $\mu_a \ll \mu_s'$ for all media used in the current work, the diffusion coefficient does not depend on light absorption, i.e. $D = 1/3\mu_s'$ [9, 10, 16].

As shown in Supplementary Fig. 6, considering a pencil beam (i.e. $I(\vec{r},\lambda) = \delta(\vec{r})$) incident on tissue with an angle of θ_i with respect to the normal direction of the surface, the light fluence can

be approximated by the illumination from two isotropic dipole sources [11, 17] and is governed by the effective light attenuation coefficient $\mu_{eff} = \sqrt{3\mu_a\mu_s'}$ and the diffusion coefficient D [17-19]:

$$\Phi_i(\vec{r},\lambda) = \gamma(\lambda)\left(\frac{1}{4\pi D(\lambda)|\vec{r}-\overrightarrow{r_{p,\iota}}|}e^{-\mu_{eff}(\lambda)|\vec{r}-\overrightarrow{r_{p,\iota}}|} - \frac{1}{4\pi D(\lambda)|\vec{r}-\overrightarrow{r_{n,\iota}}|}e^{-\mu_{eff}(\lambda)|\vec{r}-\overrightarrow{r_{n,\iota}}|}\right) \quad , \text{(S7)}$$

where $\overrightarrow{r_{p,\iota}}$ and $\overrightarrow{r_{n,\iota}}$ are the position vectors of the positive and negative sources for the i-th fiber, respectively. Parameter $\gamma(\lambda)$ is a wavelength-dependent scaling factor.

The negative source is used to satisfy the boundary condition for light propagating into the scattering medium from a non-scattering medium [18, 19]. The two sources are symmetric about the "virtual" boundary above the physical boundary by the distance $2CD$ [20]. Parameter C accounts for the effects on the incident laser beam from refractive-index mismatch at the boundary, and is a function of the effective reflection coefficient R_{eff} [9, 21]:

$$C \triangleq \frac{1+R_{eff}}{1-R_{eff}}. \quad \text{(S8)}$$

Coefficient R_{eff} is zero and, therefore, C=1 when two media have the identical refractive index. In our application, $C = 1.04$ as determined by the refractive index mismatch between the protection glass (BK-7 optical glass) at the end of the fibers and the scattering medium.

When the diffusion coefficient D is much smaller than the absorber depth, the diffusion equation can be approximated as

$$\Phi_i(\vec{r}) = -\gamma\frac{\partial}{\partial r}\frac{1}{4\pi D|\vec{r}|}e^{-\mu_{eff}|\vec{r}|}\,dr\,, \quad \text{(S9)}$$

where wavelength dependency is omitted for simplicity. We use a Cartesian coordinate system with origin at the intersection of the line between two sources and the physical boundary ($\vec{r}$ is the vector between the origin and the absorber with distance $r = \sqrt{x^2+y^2+z^2}$). Using the relation $dr = r^{-1}zdz$, Eq. (S9) reduces to

$$\Phi_i(\vec{r}) = \gamma\frac{zdz}{4\pi D}\frac{1}{r^3}\left(1+\mu_{eff}r\right)e^{-\mu_{eff}\cdot r}\,. \quad \text{(S10)}$$

Note that $dz = 6Dcos\theta_t + 4CD$, as indicated in Supplementary Fig. 6b. Eq. (S10) is further reduced to

$$\Phi_i(\vec{r}) = \rho\frac{z}{r^3}\left(1+\mu_{eff}r\right)e^{-\mu_{eff}\cdot r}\,, \quad \text{(S11)}$$

where $\rho = \gamma\frac{3cos\theta_t+2C}{2\pi}$. It is clear from Eq. (S11) that the light fluence is related only to the wavelength-dependent μ_{eff} and independent of the diffusion coefficient. Note that the fluence is not simply a pure exponentially decaying function [18].

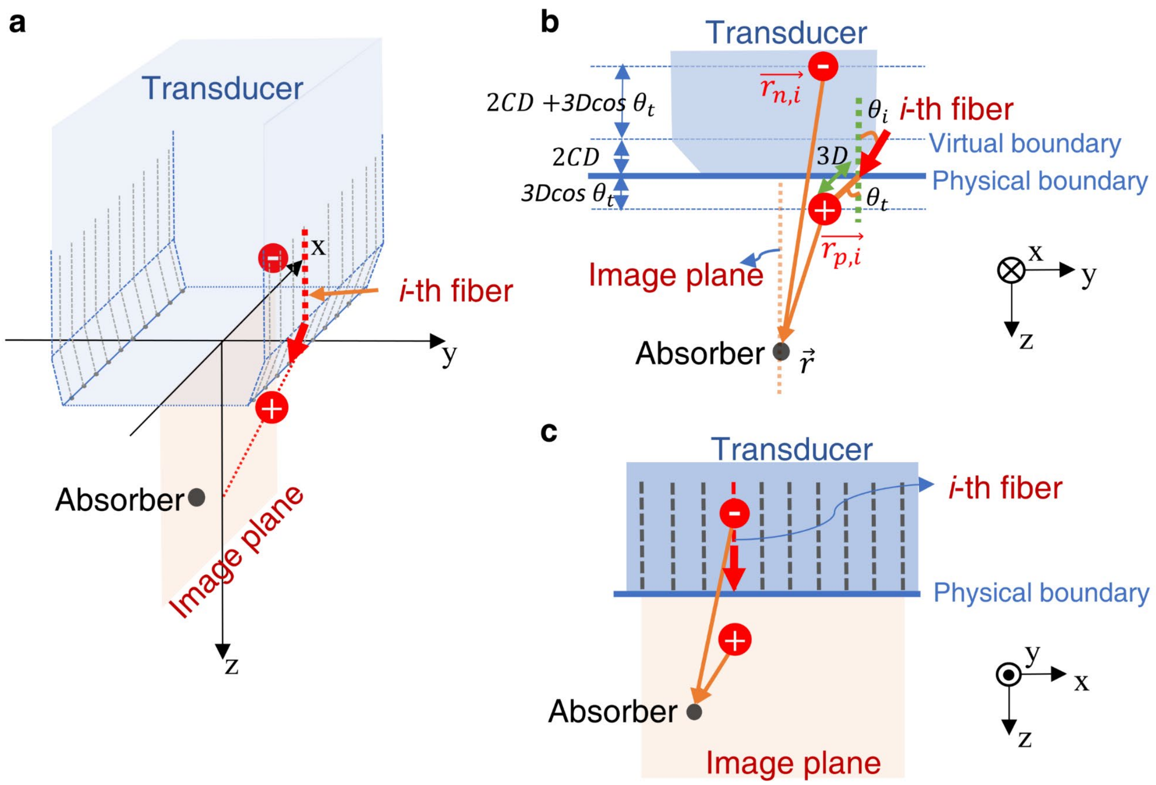

Supplementary Fig. 6: Geometry of light diffusion in the fast-sweep approach. The solution for $\Phi_i(\vec{r}, \lambda)$ can be obtained with two positive and negative sources (indicated as positive and negative signs) [9, 11, 12, 21]. **a,** 3-D schematic view. **b,** y-z plane view. **c,** x-z plane view.

Fluence (optical parameter) estimation: Since both μ_{eff} and D are functions of both μ_a and μ_s', three unknowns (γ, μ_{eff} and μ_s') must be estimated. Normalizing each fluence by the sum of all fiber fluences reduces the unknowns to μ_{eff} and μ_s', i.e.,

$$\Phi_i'(\vec{r}) = \frac{\Phi_i(\vec{r})}{\sum_{i=1}^{N_f} \Phi_i(\vec{r})} . \tag{S12}$$

Optimal estimates of these parameters can be obtained for every wavelength by minimizing the mean square error between measured PA signals $\widetilde{\Phi_i'}(\vec{r})$ for each fiber normalized to the sum of all fibers, and the normalized fluence $\Phi_i'(\vec{r})$ predicted by the diffusion model for all N_f fibers. That is

$$\hat{\mu}_{eff}, \hat{\mu}'_s = \underset{\mu_{eff}, \mu_s'}{\operatorname{argmin}} \sum_{i=1}^{N_f} \frac{(\widetilde{\Phi_i'}(\vec{r}) - \Phi_i'(\vec{r}))^2}{N_f} . \tag{S13}$$

Instead of directly solving this non-linear optimization, we use brute force search to find the optimal $\hat{\mu}_{eff}$ and $\hat{\mu}_s'$ by confining them to a reasonable region. The fluence estimate $\widehat{\Phi}_i(\vec{r})$ is obtained by substituting the estimates into either Eq. (S7) or Eq. (S11).

Spectral Correction: The final fluence-compensated PA image $P_{fc}(\vec{r},\lambda)$ becomes

$$P_{fc}(\vec{r},\lambda) = \frac{P(\vec{r},\lambda)}{\sum_{i=1}^{N_f} \hat{\Phi}_i(\vec{r},\lambda)} \cong k\Gamma\mu_a(\lambda)\,. \tag{S14}$$

Where k is a proportionality constant between measured PA signals in mV and PA pressure in Pa. We note that k is now wavelength-independent.

Real-time Implementation: The largest computational burden is brute force search to find the optimal optical parameters. Total computation complexity can be represented as $O(LMN_fN_\lambda)$ where L and M are the numbers of possible parameter states and target pixels contributing to the optimization, respectively. The number L determining search resolution can be adjusted to balance estimation accuracy and computation time. Real-time processing can be implemented with the following steps: 1) construct look-up tables containing all possible $\Phi_i'(\vec{r},\lambda)$ and store them in temporary/permanent computer storage, and 2) find an optimal solution by comparing the tables and measurement $\widetilde{\Phi_i'}(\vec{r},\lambda)$ over L in parallel.

The computation time using a GPU processor (NVIDIA GeForce RTX 2080 Ti) is **23 ms** for estimating optical parameters (9 wavelengths) of a pixel position, i.e. much faster than the 200 ms for a full spectroscopic frame. The Matlab code for real-time fluence estimation is available in the Supplementary Data and Software Library.

Supplementary Note 7. Wavelength-dependent fluence estimation in 1%, 2%, and 4% Intralipid

To confirm that optical fluence can be estimated using the diffusion approximation applied to PA data from the fast-sweep optical-fiber configuration, we conducted a set of experiments using human black hairs immersed in Intralipid solutions of different concentration. The Intralipid 20% IV fat emulsion (See Methods) is used as a tissue-mimicking optical scattering medium. Hair serves as a point-like optical absorber creating the PA signal used to estimate fluence variations as a function of wavelength. Three different concentrations, 1%, 2%, and 4% were prepared by diluting the original Intralipid 20% solution with de-ionized water in v/v.

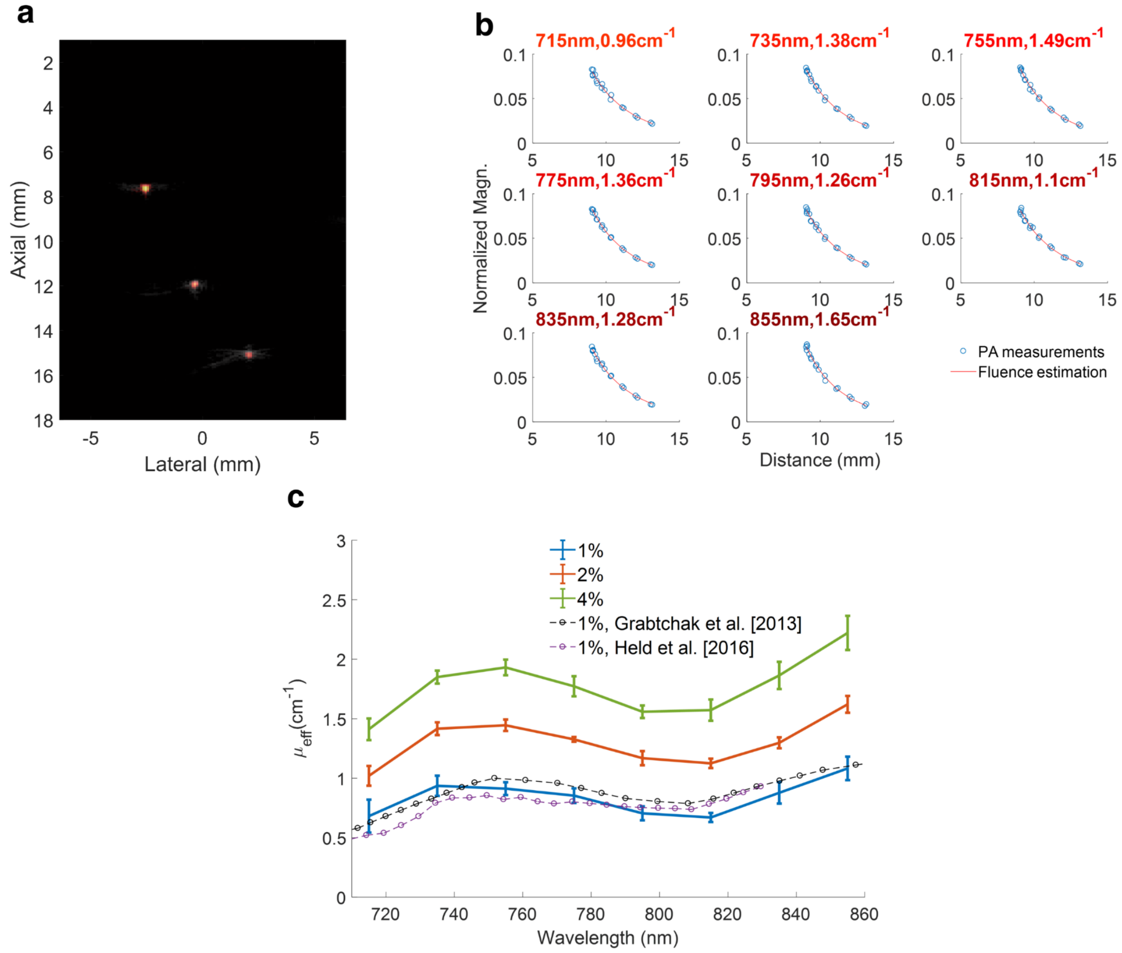

Supplementary Fig. 7: a, Σλ-PA image of three hairs in diluted 2% Intralipid solution. **b,** Using the hair at the shallowest depth (i.e., at 7.5 mm) for fluence estimation, the measured PA signals in the 2% solution are presented for all fibers (marked as blue circles) along with the estimated fluence (red solid lines) as a function of fiber-absorber distance (defined by the distance between a fiber and the target). For all panels, 8 different laser wavelengths ranging from 715 to 855 nm with an increment of 20 nm were evaluated. **c,** Estimated μ_{eff} (n=10) for 1%, 2%, and 4% Intralipid solutions as a function of wavelength. For comparison, two reported μ_{eff} from the literature for a 1% Intralipid solution are plotted together (circled dash lines) [18, 22]. The μ_{eff} reported in [18] was for a 1.2% solution and we scaled it to 1% for comparison. The estimated μ_{eff} increases with Intralipid concentration as expected, varying approximately proportional to the square root of μ_s' when $\mu_a << \mu_s'$. Clearly, the estimated μ_{eff} in 1% Intralipid solution is consistent with results from the literature [18, 22, 23].

Supplementary Note 8. Ex *vivo* fluence compensation results in chicken breast

To mimic the clinical protocol of guiding and quantifying nanoparticle injection for molecular diagnostics and therapeutics during an interventional procedure, we demonstrated fluence compensation by injecting GNRs into chicken breast. After GNR injection and needle pull out, the resulting B-mode and Σλ-PA images are shown in Supplementary Fig. 8a. Ten different laser wavelengths ranging from 700 to 875 nm were used, where 700 nm was set at zero energy to estimate the noise level.

In contrast to the high scattering Intralipid medium shown in Fig. 3, chicken breast is used to mimic a relatively low scattering *in vivo* environment [20]. As shown in Supplementary Fig. 8b, the estimated μ_s' is typically under 2 cm^{-1} and decreases monotonically with wavelength, generally consistent with results reported in [20]. Because μ_s' is small, the mean free path and diffusion coefficient are not small compared to the absorber depth. In contrast to Intralipid results where the laser fluence is mainly determined by μ_{eff}, the laser fluence in chicken breast depends on both estimates of μ_s' and μ_{eff}.

The measured spectra of GNRs were evaluated at three different lateral positions shown in the Σλ-PA image in Supplementary Fig. 8a. In each case, fluence compensation can effectively improve the GNR spectrum even though wavelength-dependent fluence variations are modest in this medium compared to the Intralipid solutions.

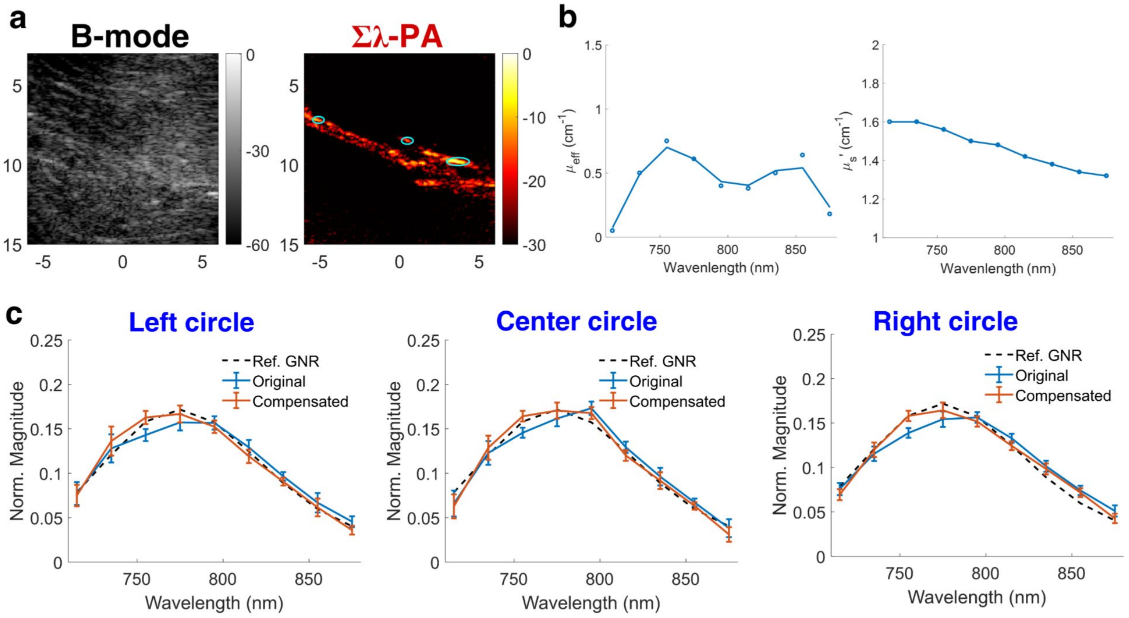

Supplementary Fig. 8: *Ex vivo* fluence compensation results of spectroscopic PAUS imaging for GNR injection in chicken breast. **a,** B-mode and Σλ-PA images. **b,** The estimated effective attenuation coefficient (μ_{eff}, left) and reduced scattering coefficient (μ_s', right) as a function of wavelength. Both are in units of cm^{-1}. In the left panel, spline-based spatial smoothing is applied to the measured μ_{eff} (marked as circles) to produce the limited-data approximation of the function (solid line). **c,** Measured GNR spectra (normalized to the area under the spectrum) were evaluated at three different positions indicated by circles in the Σλ-PA image, statistically expressed as mean ± standard deviation. Left, middle, and right panels are the results corresponding to left, middle, and right circles. For each panel, the spectrum after fluence compensation (red solid line) is compared to the uncompensated spectrum (blue line) and the reference UV-VIS result (dashed line).

<u>Supplementary Note 9</u>. Motion estimation using PatchMatch

Ultrasound speckle tracking has been extensively used to estimate tissue motion [24], whether static or dynamic, and is the core signal processing method in all of US elastography [25]. It calculates all spatial components of tissue displacements from the speckle similarity between the original image frame and the frame after induced motion and deformation [26]. The most common method is a specific form of block matching, which can be realized with the multi-dimensional cross correlation function or sum of absolute differences (SAD) processing using either envelope-detected signals (phase-insensitive tracking) or complex signals (phase-sensitive tracking) [27]. Phase-sensitive tracking is preferred because tracking accuracy has been shown to be much better when phase information is included [27].

Speckle tracking produces high-resolution motion estimates using multi-dimensional correlation functions computed at every image pixel. When the frame rate is high, however, these computations are extensive and it is hard to produce truly real-time estimates. Recently, we introduced an algorithm based on a randomized search called PatchMatch [28] to speed up processing for near real-time and truly real-time implementation [29]. We showed that PatchMatch performance is comparable to that of an extensive search while the computation load is significantly reduced by at least 10 times [29]. In addition, PatchMatch implicitly imposes smoothness on displacement estimates in adjacent pixels, which is robust against speckle decorrelation when speckle similarity is corrupted by out-of-plane motion, tissue deformation, low SNR, and other physical effects [29].

As shown here, there are three major steps in PatchMatch: initialization, propagation, and random search [28]. Take a kernel (indicated as a blue cube) in the reference volumetric image as an example. The 3-D case is presented here as the most general form of speckle tracking, but in the present study only 2-D tracking was performed on B-Mode image loops. This kernel and its spatially closest neighbors (indicated with different colors, Supplementary Fig. 9a) are assigned to the uniformly-distributed random positions in the target volumetric image (Supplementary Fig. 9b). In practice, such random assignment is confined to a pre-defined search region according to *a priori* knowledge of maximum tissue motion. The similarity between the individual pair in reference and target images is measured using the NCC (defined in Eq. (S.1)). By comparing the NCC values of all neighbors, the kernel propagates to the neighbor with maximum NCC value (Supplementary Fig. 9c). To avoid falling into a local maximum, additional search is performed by randomly selecting other positions (Supplementary Fig. 9d), confined to a pre-defined search region. In this study, six random positions are empirically determined. Last, the final position in the target image is determined by calculating the maximum NCC among all seven positions (Supplementary Fig. 9e). By iterating between propagation and random search, the final positions for all kernels converge efficiently (Supplementary Fig. 9f). In practice, iteration is performed in windshield-wiper order, i.e., from left/top to right/down and then scanned backward. Thus, two iterations complete one scan cycle. Accordingly, 2-D displacements between two US images are estimated. We have shown that 4 iterations are enough to obtain reliable estimates [29].

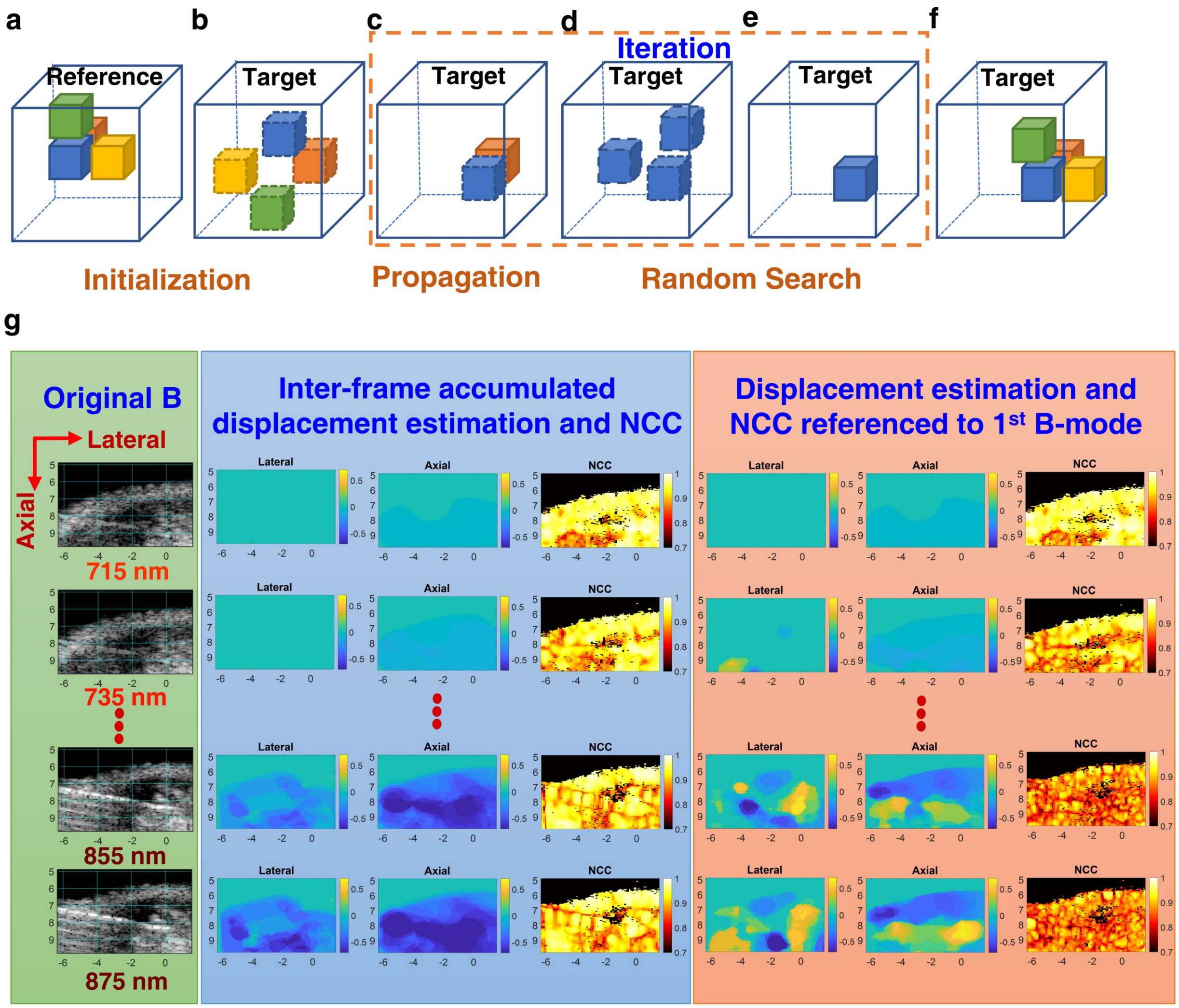

Supplementary Fig. 9. Schematic flow chart of PatchMatch for multi-dimensional displacement estimation (3-D estimation is shown here as the most general case – only 2-D tracking was performed in this study) [28, 29]. Taking a kernel (indicated as a blue cube) in the reference image as an example, **a,** six closest neighbors surrounding this kernel (only three are displayed) are indicated with different colors. **b,** The positions of the kernel and its neighbors in the target image are randomly assigned. The similarity between individual pairs in reference and target images (i.e., identical color cubes) is measured. **c,** The kernel propagates to the neighborhood having the maximum similarity. **d,** Other random positions are chosen to avoid a local maximum. **e,** Determine the final position by comparing the similarity of all candidates in (d). **f,** Iteration is performed between (c)-(e) to find the positions of all kernels. It has been shown that 4 iterations are sufficient in ultrasound motion estimation. Accordingly, displacements between two images are estimated. **g,** *in vivo* PatchMatch-based speckle tracking results of needle guidance in a mouse. Uncompensated B-mode images associated with the laser emission from 715 to 875 nm are shown in the left column. Motion estimation (i.e., lateral and axial displacement components) between two successive B-mode images (i.e., interframe or inter-wavelength estimation, middle column) is compared with that using the first B-mode (i.e., at 700 nm wavelength) as a reference (right column). Note that accumulated displacement is required for interframe estimation. As an index of tracking reliability, the NCC map for each tracking result is also presented.

Supplementary Fig. 9g shows *in vivo* results using PatchMatch-based speckle tracking on the 10 B-mode images corresponding to 10 laser wavelengths ranging from 700 to 875 nm. As mentioned earlier, we disabled 700 nm laser emission to estimate the noise level but still acquired B-mode images at this wavelength for tracking purposes. Original B-mode images corresponding to 715 nm to 875 nm are shown in the left column (same as in Fig. 4). When comparing different B-mode images, it is clear that speckle decorrelation is significant because of out-of-plane motion of a needle. Interframe (or equivalently, inter-wavelength) displacement estimation results (i.e., estimation between two successive images, middle column) are compared to the results using the first image as a reference (right column). Note that interframe estimation requires accumulation of all previous paired displacement estimates. Lateral and axial displacement components are shown in units of mm. Positive values for lateral/axial components represent the right/down direction, respectively. The NCC map for each tracking result is also shown as a reliability index. In the first two images, the two estimation approaches are similar because speckle decorrelation is small. However, for 855 and 875 nm, last-to-first frame displacement estimation exhibits larger errors as both lateral and axial components have diverse motion directions. This is because speckle decorrelation (mostly contributed by out-of-plane motion) is significant, as evidenced by the much smaller NCC values for this case. In contrast, interframe displacement estimation produces higher NCC values and uniform 2-D motion estimates under high speckle decorrelation, which is more robust than last-to-first frame estimated displacements.

The results in Supplementary Fig. 9g show that inter-wavelength (i.e., inter-frame) displacement estimation can maintain high speckle correlation with high frame rates (i.e., 50 Hz), enabling robust speckle tracking. However, if low frame rate images are used for motion compensation, as shown in Supplementary Fig. 9g where the equivalent frame rate from 1st to last wavelength is around 5 Hz, speckle decorrelation worsens displacement estimation. Therefore, motion compensation is only realistic with high frame rate imaging.

When tissue motion changes rapidly with time, the flexibility of our imaging pulse sequence enables interleaved PAUS images where the PA frame rate is 50 Hz but the US frame rate can be much higher by incorporating high speed US imaging techniques such as parallel/multibeam and compounded plane waves with a small number of angles. Increasing US frame rate to track fast motion enables more robust motion estimation while still preserving the overall 50 Hz interleaved PAUS imaging rate.

In addition to in-plane motion correction considered here, out-of-plane motion can corrupt in-plane estimates. Ideally, to account for out-of-plane motion, speckle tracking should be performed using 3-D rather than 2-D US. In this study, we used 2-D speckle tracking and estimated displacements may be corrupted by out-of-plane motion. As shown in Supplementary Fig. 9g, however, high frame rate 2D imaging can minimize the effect of out-of-plane motion on the accuracy of in-plane motion estimation.

To quantify the effects of out-of-plane motion (mainly caused by respiration) on US speckle tracking, we show below that the current frame rate is high enough so that 2-D motion estimation is robust against out-of-plane motion caused by respiration:

- The elevational beamwidth (considering 91.5 % energy concentration) defining the slice thickness in this study is 0.8 mm for a transducer with a 1.5 mm elevational width and an acoustic focus at 6 mm.

- For inter-frame motion tracking (i.e., frame time interval is 20 ms in our implementation), the maximum speed to move a target outside the elevational slice thickness is 40 mm s^{-1}.
- For normal or shallow respiratory motion (instead of deep breathing), typical displacements associated with abdominal motion are below 30 mm for shallow breathing (Table 3 of [30]). Considering that the normal breathing rate is between 12-18 per minute, the averaged speed is 6-9 mm s^{-1}.
- Considering the worst case scenario when respiratory motion is completely perpendicular to the image plane (i.e., out-of-plane motion), the averaged motion speed (i.e., 6-9 mm s^{-1}) is 4.5-7 times smaller than the maximum allowable speed (i.e., 40 mm s^{-1}). Thus, if out-of-plane motion is present, the displacement between two successive frames is well within the ultrasound elevational slice. That is, speckle decorrelation is not significant. Note that in practice, respiratory motion is generally parallel to the image plane. Therefore, the practical out-of-plane displacement will be much less than the elevation slice thickness.

Thus, we have shown that for high frame rates (i.e., 50 Hz), out-of-plane displacement mainly caused by respiration is much smaller than the slice thickness so that the speckle similarity is still relatively high enough to ensure that in-plane motion estimation is reliable. On the other hand, if the frame rate is very low (see Supplementary Fig. 9g), out-of-plane motion can produce severe speckle decorrelation corrupting in-plane motion estimation.

Real-time implementation of the proposed motion estimation and correction algorithm is demonstrated here. The computational complexity and run time associated with motion compensation using PatchMatch have been analyzed by our group (See Table 3 in [29]). This algorithm has total arithmetic operations (or equivalently, floating point operations per second (FLOPS)) that can be expressed as $\left(1 + N_i(1 + N_s)\right)(9r_kK_lK_aK_e + 3)$, where N_i is the number of iterations in PatchMatch (4 in this study); N_s is the number of random searches (1); $K_lK_aK_e$ are the lateral, axial, and elevational kernel size used for tracking (7,17,1, respectively); r_k =1 in this study. For a 2-D image with a size of 128 × 512 pixels doing speckle tracking every 20 ms, 32 GFLOPS are required. Compared to a current off-the-shelf Intel Core i9-9900KF CPU processor which has 588 GFLOPS, our motion correction potentially can easily run within 20 ms.

To demonstrate the real-time capability of the proposed motion estimation and correction algorithm, *in vivo* mouse nanoparticle injection data were processed with our developed Matlab MEX-based PatchMatch core function. All codes and data are available and described in detail in Supplementary Data and Software Library. Using the same search parameters mentioned earlier and without any GPU and parallel implementation, we have shown the measured run times over all wavelengths for a predefined region of interest (7.6 mm by 2.2 mm) are well less than 20 ms (the run time for 9 inter-wavelength motion estimates can be 11 ms) using Matlab R2019b with an Intel Core i9-9900KF CPU at 3.6 GHz.

<u>Supplementary Note 10</u>. Supplementary data and software library

To demonstrate the real-time capability of our developed PAUS imaging system, three major algorithms including PAUS beamforming, fluence estimation, and motion compensation were implemented to achieve a 50 Hz frame rate. The code and data are provided. Methods and requirements for running the programs are described in detail below.

Hardware/Software testing conditions (and requirements):

(1) Intel Core i9-9900KF CPU at 3.6 GHz
(2) NVIDIA GeForce RTX 2080Ti GPU
(3) DDR4-2666 64 GBytes RAM
(4) Matlab 2019b (on Microsoft Windows 10)

PAUS beamforming

Directly executing GPU-accelerated code “Beamforming.m” produces the final PA image (see Supplementary Fig. 10). There are four files included and described below.

rfData.mat: RF raw channel data representing PA signals from 4 optical absorbers. The dimension is 2048 (axial number of samples) × 128 (receive channels) × 20 (number of fibers) × 30 (number of frames) samples.

parameterAll.mat: Parameters required for image formation. A structure called “param” contains all parameters for system, data and reconstruction.

Beamforming.m: The main program for performing image reconstruction or beamforming. The final beamformed PA image has a dimension of 512 (axial) × 128 (lateral) pixels.

LUTGenerator.m: Subroutine to generate the look-up table for image reconstruction. After execution, the code can automatically save a table file called “PADASFastTable.mat”.

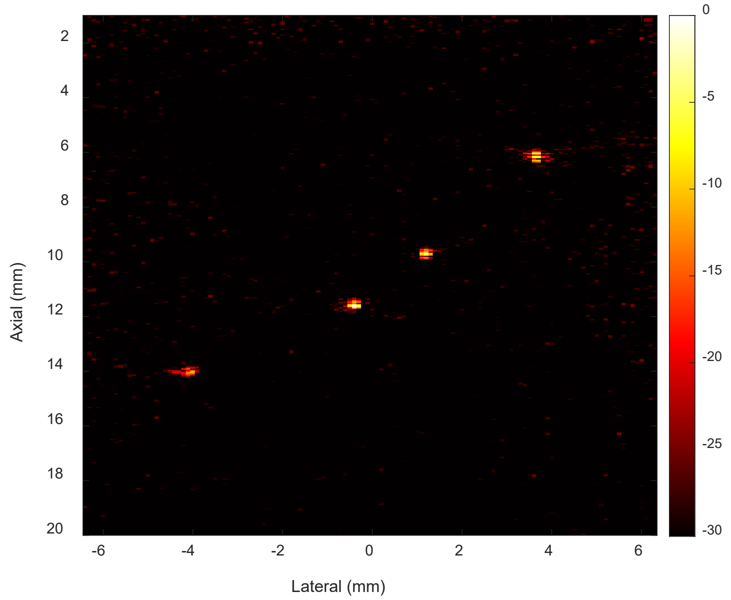

Supplementary Fig. 10. Real-time GPU-accelerated PA image. The display color scale is in dB relative to the brightest signal in the image**.**

Fluence estimation

Directly execute GPU-accelerated code "parameter2DsearchGPU.m" to produce optical fluence estimates (see Supplementary Fig. 11). Three files are included and described below.

multiWavData.mat: Beamformed experimental image data called 'totalImg' are processed. The dimension is 1024 (axial) × 128 (lateral) × 20 (number of fibers) × 1 (number of frames) × 10 (number of wavelengths) samples.

parameter2DsearchGPU.m: The main program for fluence estimation to obtain the effective attenuation coefficient (upper-left), absorption coefficient (upper-right), reduced scattering coefficient (lower-left) and free path length (lower-right).

multiWavParam.m: Parameters required for fluence compensation. A 'param' contains all parameters for system, data and reconstruction.

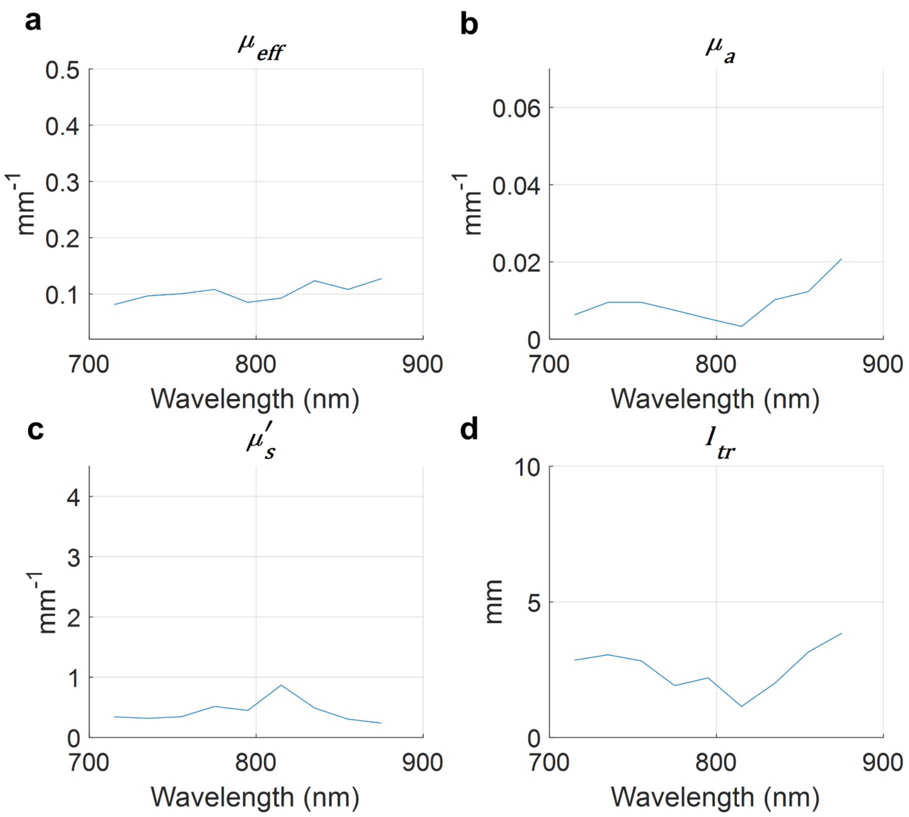

Supplementary Fig. 11. Real-time GPU-accelerated fluence estimates including: **a,** μ_{eff}; **b,** μ_a; **c,** μ_s', and **d,** l_{tr}.

Motion correction

Directly execute the code "PM_realtime.m" to produce 9 inter-wavelength motion estimates and the compensated PA image. Supplementary Fig. 12 shows representative results. Three files are included and described below.

PM_realtime.m: The main program for inter-wavelength motion estimation and compensation.

PM2DabsRT_mex.mexw64: MEX-based core function performing PatchMatch US speckle tracking.

PAUS_10frame_motion.mat: *in vivo* mouse PAUS beamformed data during nanoparticle injection, including 10 different wavelengths (the variable "pamode_all") and interleaved B modes (the variable "bmode_all"). Both data sets have a dimension of 513 (axial) × 128 (lateral) × 10 (number of wavelengths) samples. The corresponding lateral and axial extents (the variables "SL_pos" and "range", respectively) required for motion estimation are also included in this file.

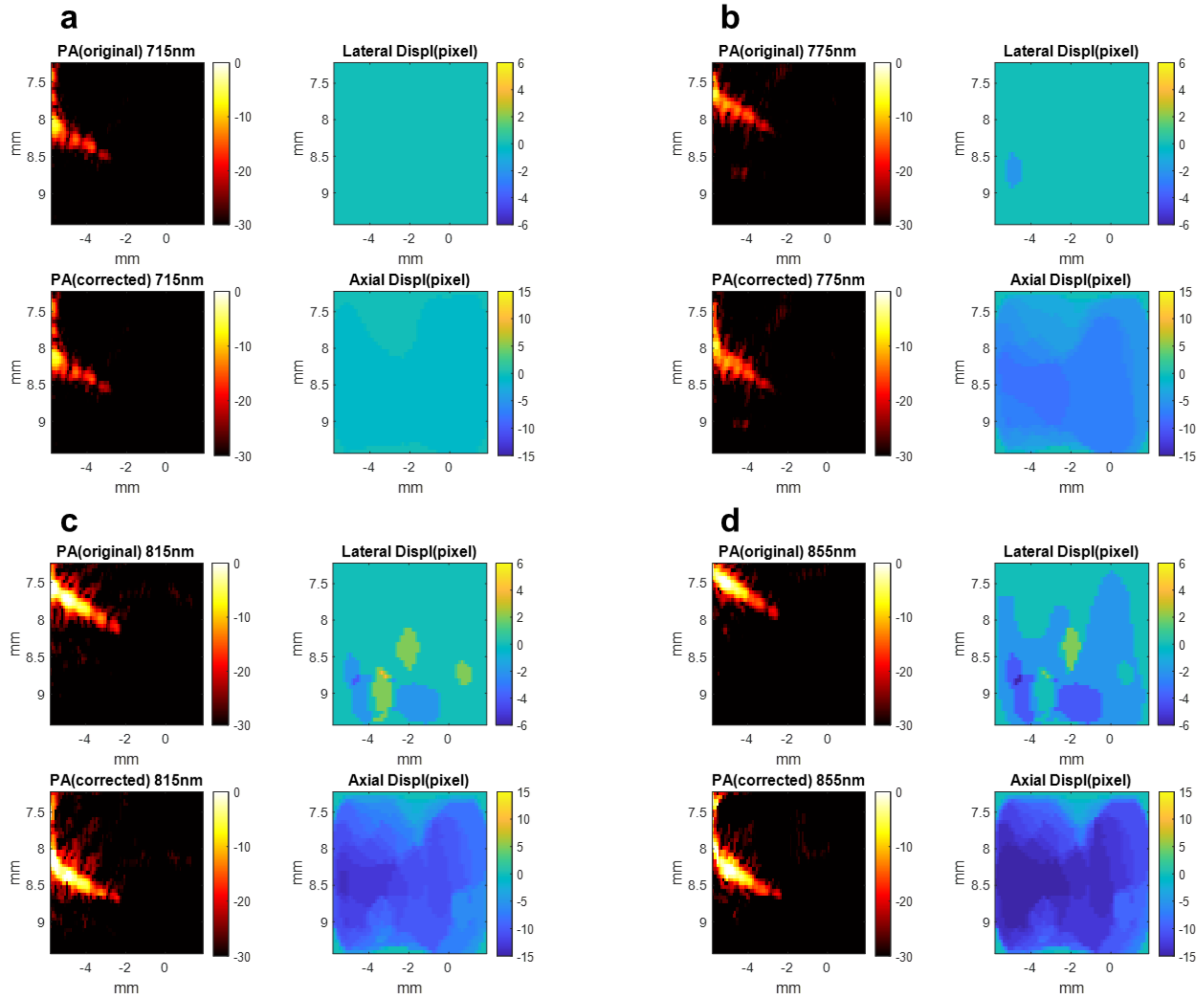

Supplementary Fig. 12. Real-time inter-wavelength motion estimates and the compensated PA image at **a,** 715 nm, 2nd frame; **b,** 775 nm, 5th frame; **c,** 815nm,7th frame; and **d,** 855nm, 9th frame. In each panel, the original PA image (upper-left) and corrected PA image (lower-left) are compared, as well as the estimated accumulated lateral (upper-right) and axial displacement (lower-right) in units of image pixels.

Supplementary References

1. Zhu, W., Liu, K, Sun, X., Wang, X., Li, Y., Cheng, L., Liu, Z. Mn2+-doped prussian blue nanocubes for bimodal imaging and photothermal therapy with enhanced performance. *ACS Appl. Mater. Interfaces* **7**(21), 11575-11582 (2015).

2. Ren, W., Qin, M., Zhu, Z., Yan, M., Li, Q., Zhang, L., Liu D., Mai, L. Activation of Sodium Storage Sites in Prussian Blue Analogues via Surface Etching. *Nano Lett.* **17**(8), 4713-4718 (2017).

3. Van Staveren, H. J., Moes, C. J. M., van Marie, J., Prahl, S. A., van Gemert, M. J. C. Light scattering in lntralipid-10% in the wavelength range of 400–1100 nm. *Appl. Opt.* **30**(31), 4507-4514 (1991).

4. Jacques, S. L. Optical properties of biological tissues: a review. *Phys. Med. Biol.* **58**, R37-R61 (2013).

5. Tzoumas, S., Nunes, A., Olefir, I., Stangl, S., Symvoulidis, P., Glasl, S., Bayer, C., Multhoff, G., Ntziachristos, V. Eigenspectra optoacoustic tomography achieves quantitative blood oxygenation imaging deep in tissues, *Nature Communications* **7**, 12121 (2016).

6. Van de Hulst, H.C. *Light Scattering by Small Particles* (Dover Publications, New York, 1981).

7. Furutsu, K., Yamada, Y. Diffusion approximation for a dissipative random medium and the applications. *Phys. Rev. E* **50**(5), 3634 -3640 (1994).

8. Ishimaru, A. *Wave propagation and scattering in random media* (Academic Press, New York, 1977).

9. Karabutov, A. A., Pelivanov, I. M., Podymova, N. B., Skipetrov, S. E., Determination of the optical characteristics of turbid media by the laser optoacoustic method. *Quantum Electronics* **29**, 215-220 (1999).

10. Grashin, P. S., Karabutov, A. A., Oraevsky, A. A., Pelivanov, I. M., Podymova, N. B., Savateeva, E. V., Solomatin, V. S. Distribution of the laser radiation intensity in turbid media: Monte-Carlo simulations, theoretical analysis and the results of optoacoustic measurements. *Quantum Electronics* **32**, 868-874 (2002).

11. Jacques, S. L. Light distributions from point, line and plane sources for photochemical reactions and fluorescence in turbid tissues. *Photochem. Photobiol*. **67**(1), 23-32 (1998).

12. Jia, M., Chen, X., Zhao, H., Cui, S., Liu, M., Liu, L., Gao, F. Virtual-source diffusion approximation for enhanced near-field modeling of photon-migration in low-albedo medium. *Opt. Express* **23**(2), 1337-1352 (2015).

13. Corngold, N., Aronson, R. Photon diffusion coefficient in an absorbing medium. *J. Opt. Soc. Am.* **16**(5), 1066-1071 (1999).

14. Pierrat, R., Greffet, J.-J., Carminati, R. Photon diffusion coefficient in scattering and absorbing media. *J. Opt. Soc. Am.* **23**(5), 1106-1110 (2006).

15. Ripoll, J., Yessayan, D., Zacharakis,G., Ntziachristos, V. Experimental determination of photon propagation in highly absorbing and scattering media. *J. Opt. Soc. Am.* **22**(3), (2005).

16. Glasston, S., Edlund, M. C. *The elements of nuclear reactor theory* (D. Van Nostrand Co., Inc., New York, 1952).

17. Farrell, T. J., Patterson, M. S., Wilson, B. A diffusion theory model of spatially resolved, steady-state diffuse reflectance for the noninvasive determination of tissue optical properties in vivo. *Med. Phys.* **19**(4), 879-888 (1992).

18. Held, K. G., Jaeger, M., Rička, J., Frenz, M., Akarçay, H. G. Multiple irradiation sensing of the optical effective attenuation coefficient for spectral correction in handheld OA imaging. *Photoacoustics* **4**(2), 70-80 (2016).

19. Ranasinghesagara, J. C., Zemp, R. J. Combined photoacoustic and oblique-incidence diffuse reflectance system for quantitative photoacoustic imaging in turbid media. *J. Biomed. Opt*. **15**(4), 046016 (2010).

20. Marquez, G., Wang, L. V., Lin, S. P., Schwartz, J. A., Thomsen, S. L. Anisotropy in the absorption and scattering spectra of chicken breast tissue. *App. Optics*, **37**(4), 798-804 (1998).

21. Wang, L. V., Wu, H.-I., *Biomedical optics: principles and imaging*. (John Wiley & Sons, 2012).

22. Grabtchak, S., Callaghan, K. B., Whelan, W. M. Tagging photons with gold nanoparticles as localized absorbers in optical measurements in turbid media. *Biomed. Opt. Express* **4**(12) 2989-3006 (2013).

23. Flock, S. T., Jacques, S. L., Wilson, B. C., Star, W. M., van Gemert, M. J. Optical properties of Intralipid: a phantom medium for light propagation studies. *Lasers Surg. Med.* **12**(5), 510-519 (1992).

24. Kaluzynski, K., Chen, X., Emelianov, S. Y., Skovoroda, A. R., O'Donnell, M. Strain rate imaging using two-dimensional speckle tracking. *IEEE Trans. Ultrason. Ferroelectr. Freq. Contr.* **48**(4), 1111-1123 (2001).

25. D'Hooge, J., Konofagou, E., Jamal, F., Heimdal, A., Barrios, L., Bijnens, B., Thoen, J., Van de Werf, F., Sutherland, G., Suetens, P. Two-dimensional ultrasonic strain rate measurement of the human heart in vivo. *IEEE Trans. Ultrason. Ferroelectr. Freq. Contr.* **49**(2), 281-286 (2002).

26. Lubinski, M. A., Emelianov, S. Y., O'Donnell, M. Speckle tracking methods for ultrasonic elasticity imaging using short-time correlation. *IEEE Trans. Ultrason. Ferroelectr. Freq. Contr.* **46**(1), 82-96 (1999).

27. Viola, F., Walker, W. F. A comparison of the performance of time-delay estimators in medical ultrasound. *IEEE Trans. Ultrason. Ferroelectr. Freq. Contr.* **50**(4), 392-401 (2003).

28. Barnes, C., Shechtman, E., Finkelstein, A., Goldman, D. B. PatchMAtch: a randomized correspondence algorithm for structural image editing. *ACM Transactions on Graphics* (Proc. SIGGRAPH) **28**(3), (2009).

29. Jeng, G.-S., Zontak, M., Parajuli, N., Lu, A., Ta, K., Sinusas, A. J., Duncan, J. S., O'Donnell, M. Efficient two-pass 3-D speckle tracking for ultrasound imaging. *IEEE Access* **6**, 17415-17428 (2018).

30. Keall, P. J. et al. The management of respiratory motion in radiation oncology report of AAPM Task Group 76. *Med. Phys.* **33**(10), 3874–3900 (2006).
