# Supplementary material for: Real-time interleaved spectroscopic photoacoustic and ultrasound (PAUS) scanning with simultaneous fluence compensation and motion correction
Source: Nat Commun. 2021 Jan 29;12:716. doi: 10.1038/s41467-021-20947-5 (PMC7846772; doi:10.1038/s41467-021-20947-5)
Supplement: Supplementary file 3 — Description of Additional Supplementary Files [file 41467_2021_20947_MOESM3_ESM.pdf]

Description of Additional Supplementary Files

File: Supplementary Movie 1
Description: Real-time ex vivo spectroscopic PAUS imaging for needle guidance with nanoparticle injection at a 50-Hz frame rate showing three stages: needle insertion into chicken breast, GNR injection, and needle pullout. For each stage, PA images are acquired at a single wavelength (775 nm), followed by a 10-wavelength sweep ranging from 700 to 875 nm. The laser sequence (right) is shown synchronized with the real-time image (left).

File: Supplementary Movie 2
Description: Real-time in vivo mouse spectroscopic PAUS imaging for needle guidance with nanoparticle injection at a 50-Hz frame rate. PA images are acquired at a single wavelength (775 nm), followed by a 10-wavelength sweep ranging from 700 to 875 nm. The laser sequence (right) is shown synchronized with the real-time image (left).

File: Supplementary Software 1
Descripiton: PAUS beamforming
Beamforming.m (main program): The main program for performing image reconstruction. The final beamformed PA image has a dimension of 512 (axial) × 128 (lateral) pixels.

File: Supplementary Software 2
Descripiton: PAUS beamforming
parameterAll.mat: Parameters required for image formation. A structure called "param" contains all parameters for system, data and reconstruction.

File: Supplementary Software 3
Descripiton: PAUS beamforming
LUTGenerator.m: Subroutine to generate the look-up table for image reconstruction. After execution, the code can automatically save a table file called "PADASFastTable.mat".

File: Supplementary Software 4
Description: Fluence estimation
parameter2DsearchGPU.m (main program): To obtain the effective attenuation coefficient, absorption coefficient, reduced scattering coefficient and free path length.

File: Supplementary Software 5
Description: Fluence estimation
multiWavParam.m: Parameters required for fluence compensation. A 'param' contains all parameters for system, data and reconstruction.

File: Supplementary Software 6
Description: Motion correction
PM_realtime.m (main program): The main program for inter-wavelength motion estimation and compensation.

File: Supplementary Software 7
Description: Motion correction
PM2DabsRT_mex.mexw64: MEX-based core function performing PatchMatch US speckle tracking.
Box 355061 1705 NE Pacific St. Seattle, WA 98195-5061
206.685.2002 fax 206.685.3300 bioechr@u.washington.edu depts.washington.edu/bioep. 5

File: Supplementary Data 1
Description: rfData.mat: RF raw channel data representing PA signals from 4 optical absorbers. The dimension is 2048 (axial number of samples) × 128 (receive channels) × 20 (number of fibers) × 30 (number of frames) samples.

File: Supplementary Data 2
Description: multiWavData.mat: Beamformed experimental image data called 'totalImg' are processed. The dimension is 1024 (axial) × 128 (lateral) × 20 (number of fibers) × 1 (number of frames) × 10 (number of wavelengths) samples.

File: Supplementary Data 3
Description: PAUS_10frame_motion.mat: in vivo mouse PAUS beamformed data during nanoparticle injection,including 10 different wavelengths (the variable "pamode_all") and interleaved B modes (the variable "bmode_all"). Both data sets have a dimension of 513 (axial) × 128 (lateral) × 10 (number of wavelengths) samples. The corresponding lateral and axial extents (the variables "SL_pos" and "range", respectively) required for motion estimation are also included in this file.
