# Supplementary material for: Real-time interleaved spectroscopic photoacoustic and ultrasound (PAUS) scanning with simultaneous fluence compensation and motion correction
Source: Nat Commun. 2021 Jan 29;12:716. doi: 10.1038/s41467-021-20947-5 (PMC7846772; doi:10.1038/s41467-021-20947-5)
Supplement: Supplementary file 16 — Reporting Summary [file 41467_2021_20947_MOESM16_ESM.pdf]

nature research

Corresponding author(s): Ivan Pelivanov

Last updated by author(s): Sep 2, 2020

Reporting Summary

Nature Research wishes to improve the reproducibility of the work that we publish. This form provides structure for consistency and transparency in reporting. For further information on Nature Research policies, see our Editorial Policies and the Editorial Policy Checklist.

Statistics

For all statistical analyses, confirm that the following items are present in the figure legend, table legend, main text, or Methods section.

n/a	Confirmed	
☐	☒	The exact sample size (*n*) for each experimental group/condition, given as a discrete number and unit of measurement
☐	☒	A statement on whether measurements were taken from distinct samples or whether the same sample was measured repeatedly
☐	☒	The statistical test(s) used AND whether they are one- or two-sided<br>*Only common tests should be described solely by name; describe more complex techniques in the Methods section.*
☐	☒	A description of all covariates tested
☐	☒	A description of any assumptions or corrections, such as tests of normality and adjustment for multiple comparisons
☐	☒	A full description of the statistical parameters including central tendency (e.g. means) or other basic estimates (e.g. regression coefficient) AND variation (e.g. standard deviation) or associated estimates of uncertainty (e.g. confidence intervals)
☒	☐	For null hypothesis testing, the test statistic (e.g. *F*, *t*, *r*) with confidence intervals, effect sizes, degrees of freedom and *P* value noted<br>*Give P values as exact values whenever suitable.*
☒	☐	For Bayesian analysis, information on the choice of priors and Markov chain Monte Carlo settings
☒	☐	For hierarchical and complex designs, identification of the appropriate level for tests and full reporting of outcomes
☒	☐	Estimates of effect sizes (e.g. Cohen's *d*, Pearson's *r*), indicating how they were calculated

Our web collection on statistics for biologists contains articles on many of the points above.

Software and code

Policy information about availability of computer code

Data collection	All data were collected using (1) a customized pulsed laser system with custom software (Version: LCC300T1Factory, Laser-Export, Russia) controlling laser emissions; (2) a customized fiber-optic delivery system with custom software (Version: Kangoo, TEM-Messtechnik, Germany); and (3) a commercial ultrasound scanner (Vantage, Verasonics, USA) where Matlab-based scripts (R2017b, MathWorks, USA) was used for ultrasonic array data acquisition and overall control of the PAUS scanner.
Data analysis	All data were analyzed using Matlab (R2019b, MathWorks, MA, USA).

For manuscripts utilizing custom algorithms or software that are central to the research but not yet described in published literature, software must be made available to editors and reviewers. We strongly encourage code deposition in a community repository (e.g. GitHub). See the Nature Research guidelines for submitting code & software for further information.

Data

Policy information about availability of data

All manuscripts must include a data availability statement. This statement should provide the following information, where applicable:

- Accession codes, unique identifiers, or web links for publicly available datasets
- A list of figures that have associated raw data
- A description of any restrictions on data availability

The authors declare that all data from this study are available within the Article and its Supplementary Information. We share some experimental raw datasets and provide all processing routines in Supplementary Data and Software Library with detailed instructions on how to run the scripts. The full package of raw datasets generated during and/or analyzed during the current study is available from the corresponding author on reasonable request.

Field-specific reporting

Please select the one below that is the best fit for your research. If you are not sure, read the appropriate sections before making your selection.

- [x] Life sciences
- [ ] Behavioural & social sciences
- [ ] Ecological, evolutionary & environmental sciences

For a reference copy of the document with all sections, see nature.com/documents/nr-reporting-summary-flat.pdf

Life sciences study design

All studies must disclose on these points even when the disclosure is negative.

Sample size	Statistical analysis was not required in this study because the study was focused on demonstrating the imaging capabilities of a new imaging method
Data exclusions	No data were excluded for the analysis/processing described in the paper
Replication	All attempts at replication of experimental results were successful. Inaccuracies are reported in terms of Standard Deviations where applicable.
Randomization	This study was focused on demonstrating the echnical capabilities of a new imaging method. It did not include clinical data and statistical analysis was not required. Thus, 'Randomization' of samples/animals is not applicable for this study.
Blinding	This study was performed by a professional research group requiring certain skills in the area of research. Thus, 'Blinding' is not applicable for this study.

Reporting for specific materials, systems and methods

We require information from authors about some types of materials, experimental systems and methods used in many studies. Here, indicate whether each material, system or method listed is relevant to your study. If you are not sure if a list item applies to your research, read the appropriate section before selecting a response.

Materials & experimental systems

n/a	Involved in the study	
x		Antibodies
x		Eukaryotic cell lines
x		Palaeontology and archaeology
	x	Animals and other organisms
x		Human research participants
x		Clinical data
x		Dual use research of concern

Methods

n/a	Involved in the study	
x		ChIP-seq
x		Flow cytometry
x		MRI-based neuroimaging

Animals and other organisms

Policy information about studies involving animals; ARRIVE guidelines recommended for reporting animal research

Laboratory animals	8-week-old nude female mouse (nu/nu, strain code: 088, Charles River Laboratories, USA)
Wild animals	N/A
Field-collected samples	N/A
Ethics oversight	All studies involving small animals (mice) were performed with approval of the Institutional Animal Care and Use Committee of the University of Washington (Proto201600723:4211-03)

Note that full information on the approval of the study protocol must also be provided in the manuscript.
